# Regulation of the Tyrosine Phosphorylation of Phospholipid Scramblase 1 in Mast Cells That Are Stimulated through the High-Affinity IgE Receptor

**DOI:** 10.1371/journal.pone.0109800

**Published:** 2014-10-07

**Authors:** Asma Kassas, Ivan C. Moura, Yumi Yamashita, Jorg Scheffel, Claudine Guérin-Marchand, Ulrich Blank, Peter J. Sims, Therese Wiedmer, Renato C. Monteiro, Juan Rivera, Nicolas Charles, Marc Benhamou

**Affiliations:** 1 INSERM U1149, Faculté de Médecine Xavier Bichat, Paris, France; 2 University Paris-Diderot, Sorbonne Paris Cité, Laboratoire d’excellence INFLAMEX, DHU FIRE, Paris, France; 3 Laboratory of Molecular Immunogenetics, Molecular Immunology and Inflammation Branch, NIAMSD, NIH, Bethesda, Maryland, United States of America; 4 Department of Medicine, University of Rochester School of Medicine and Dentistry, Rochester, New York, United States of America; Cornell University, United States of America

## Abstract

Engagement of high-affinity immunoglobulin E receptors (FcεRI) activates two signaling pathways in mast cells. The Lyn pathway leads to recruitment of Syk and to calcium mobilization whereas the Fyn pathway leads to phosphatidylinositol 3-kinase recruitment. Mapping the connections between both pathways remains an important task to be completed. We previously reported that Phospholipid Scramblase 1 (PLSCR1) is phosphorylated on tyrosine after cross-linking FcεRI on RBL-2H3 rat mast cells, amplifies mast cell degranulation, and is associated with both Lyn and Syk tyrosine kinases. Here, analysis of the pathway leading to PLSCR1 tyrosine phosphorylation reveals that it depends on the FcRγ chain. FcεRI aggregation in Fyn-deficient mouse bone marrow-derived mast cells (BMMC) induced a more robust increase in FcεRI-dependent tyrosine phosphorylation of PLSCR1 compared to wild-type cells, whereas PLSCR1 phosphorylation was abolished in Lyn-deficient BMMC. Lyn association with PLSCR1 was not altered in Fyn-deficient BMMC. PLSCR1 phosphorylation was also dependent on the kinase Syk and significantly, but partially, dependent on detectable calcium mobilization. Thus, the Lyn/Syk/calcium axis promotes PLSCR1 phosphorylation in multiple ways. Conversely, the Fyn-dependent pathway negatively regulates it. This study reveals a complex regulation for PLSCR1 tyrosine phosphorylation in FcεRI-activated mast cells and that PLSCR1 sits at a crossroads between Lyn and Fyn pathways.

## Introduction

High-affinity receptors for IgE (FcεRI) expressed on mast cells promote, after their aggregation by IgE and antigen, the release of preformed mediators stored in cytoplasmic granules and of newly synthesized lipid mediators and cytokines [1]. Engagement of FcεRI leads to the activation of at least two signaling pathways. One is initiated by the tyrosine kinase Lyn [2] and leads to recruitment of another tyrosine kinase, Syk, to the receptor and to activation of the signaling complex recruited by the protein adaptor LAT [3], resulting in calcium mobilization [4]. The other pathway, initiated by the tyrosine kinase Fyn [4], leads to phosphatidylinositol 3-kinase recruitment [4], [5]. Both pathways cooperate to determine the extent of degranulation and of cytokine and lipid inflammatory mediator production. It has been demonstrated that the Lyn-initiated pathway negatively regulates the Fyn-initiated pathway through recruitment of the kinase Csk [6]. Since the FcεRI-dependent cell activation combines these pathways into one coherent signal, mapping of their connections is an important task that remains to be completed to fully understand signal integration.

Recently, we reported that phospholipid scramblase 1 (PLSCR1) is phosphorylated on tyrosine after aggregation of FcεRI on mast cells [7]. PLSCR1 is a multi-function protein. It was originally identified based on its capacity to accelerate transbilayer migration of phospholipids upon interaction with calcium, thereby collapsing the lipid asymmetry existing between inner and outer leaflets of plasma membranes [8], [9]. Activation of scrambling leads to increased cell surface exposure of phosphatidylserine and other aminophospholipids. This has been implicated in the recognition of apoptotic cells by phagocytes and in the cell surface expression of procoagulant activity by activated platelets and perturbed endothelium [10], [11]. Interestingly, activated mast cells also demonstrate transient exposure of phosphatidylserine [12], [13]. However, studies with knock-out mice questioned the involvement of PLSCR1 alone in phospholipid scrambling [14], [15]. Recently, several reports have implicated the Ca^2+^-activated ion channels belonging to the TMEM16 family in phospholipid scrambling induced by a calcium ionophore [16]–[18]. By contrast, phospholipid scrambling following caspase activation during apoptosis was shown to be promoted by Xkr8, a putative transporter [19]. Therefore, depending on the triggering signal, phospholipid scrambling now appears to result from a variety of alternative mechanisms, in which the specific role of plasma membrane PLSCR1 remains to be resolved. In addition to its putative role in mediating transbilayer movement of plasma membrane phospholipids that accompanies PS exposure at the cell surface, there is now also considerable evidence that: i) PLSCR1 serves as a signaling intermediate for the Epidermal Growth Factor (EGF) receptor promoting optimal activation of p60c-Src [20], [21]; ii) PLSCR1 contains a nuclear localisation signal domain that mediates nuclear trafficking of the unpalmitoylated form of the protein [22], [23]; iii) *de* *novo* synthesis of PLSCR1 is induced by interferon-α (IFNα) and results in its nuclear trafficking and binding to chromosomal DNA [23]–[25]. In this setting, PLSCR1 may serve as a transcription factor since it amplifies the expression of IFNα/β-stimulated genes [26] and promotes the transcription of the inositol 1, 4, 5-trisphosphate receptor gene [27]; iv) PLSCR1 potentiates granulopoiesis by prolonging expansion of granulocyte precursors presumably through its role in transcriptional regulation [15]; v) Expression of PLSCR1 has been shown to be tumor suppressive, and its level of expression in bone marrow cells to correlate with long-term survival in acute myelogenous leukemia, whereas mutations affecting PLSCR1 appear to promote the leukemogenic potential of myeloid progenitors [28]–[31]; vi) PLSCR1 regulates compensatory endocytosis in neuroendocrine cells [32]; vii) PLSCR1 is capable of potentiating a select set of mast cell responses following FcεRI aggregation [33]. In this study, we observed that endogenous expression of PLSCR1 in RBL-2H3 mast cells doubles VEGF production and the degranulation response to FcεRI engagement as compared to PLSCR1-knock-down RBL-2H3 cells, without any detectable impact on MCP-1 production and release of arachidonic acid metabolites. In PLSCR1-knocked-down RBL-2H3 cells the LAT-PLCγ-calcium axis initiated by Lyn was inhibited [33]. Interestingly, Lyn was found to colocalize with PLSCR1 at the cell membrane and to co-precipitate with it. Syk, which is downstream of Lyn activation, was also found to interact with PLSCR1 [33].

Whereas the kinase responsible for the tyrosine phosphorylation of PLSCR1 after engagement of the EGF receptor has been identified as p60c-Src [20], [21], and as c-Abl after induction of apoptosis [34], the regulatory pathway leading to PLSCR1 tyrosine phosphorylation after FcεRI aggregation remains to be elucidated. The present study was carried out to identify this pathway. We found that the Lyn and Fyn pathways cooperate in FcεRI-dependent tyrosine phosphorylation of PLSCR1. Indeed, PLSCR1 phosphorylation was dependent on FcRγ chain, on Lyn and on Syk and partially on intracellular calcium mobilization, suggesting direct involvement of both kinases through a direct association with PLSCR1, and indirectly as a result of calcium mobilization. We also provide evidence that Fyn negatively regulates PLSCR1 tyrosine phosphorylation.

## Materials and Methods

### Ethics statement

Mice were maintained and used in accordance with NIH guidelines and Animal Study Proposals approved by the NIAMS Animal Care and Use Committee (ASP number AO12-11-01).

### Antibodies and reagents

The anti-DNP monoclonal IgE DNP-48 [35] was a kind gift of Dr. Reuben Siraganian (NIH, Bethesda, MD). The monoclonal anti-rat PLSCR1 antibody 129.2 was produced in the laboratory [7], [36]. Monoclonal anti-mouse PLSCR1 antibody 1A8 has been described elsewhere [20]. Anti-Lyn and anti-Fyn polyclonal antibodies were from Santa-Cruz Biotech (Santa-Cruz, CA). Anti-phosphotyrosine monoclonal antibody 4G10 was used as a culture supernatant of the 4G10 hybridoma or as horseraddish peroxidase (HRP)-coupled antibody (Calbiochem, La Jolla, CA). HRP-labeled secondary antibodies (rabbit anti-mouse and goat anti-rabbit) were from Sigma-Aldrich (St-Louis, MO). Cell culture medium (D-MEM and E-MEM), fetal calf serum and other culture reagents were from Gibco-BRL-Life Technologies (Gaithersburg, MD). Triton X-100 and sodium dodecyl sulfate (SDS), bovine serum albumin (BSA), DNP-human serum albumin (DNP-HSA), proteases and phosphatases inhibitors (leupeptin and aprotinin, NaF and Na_3_VO_4_) were from Sigma-Aldrich (St-Louis, MO). G418 was from PAA Laboratories (Les Mureaux, France).

### Cells and cell lines

The RBL-2H3 [37] or the β-hexosaminidase high-releaser subclone 9 of this cell line were maintained in culture as described [13]. The Syk-deficient RBL-2H3 variant and the Syk reconstituted clone have been described elsewhere [38] and were generously provided by Dr. Reuben P. Siraganian (NIDCR, NIH, Bethesda, MD). The Syk reconstituted clone was maintained in selective medium containing 1 mg/ml G418.

RBL-2H3 transfectants expressing either the wild-type FcαRI or the mutated R209L FcαRI have been described elsewhere and express equivalent levels of the transfected receptor [39]. Whereas the wild-type FcαRI does associate with endogenous FcRγ chain, the mutated R209L FcαRI does not, as determined by co-immunoprecipitation experiments [39]. These transfectants were maintained in selective medium containing 1 mg/ml G418.

Bone marrow-derived mast cells (BMMC) were obtained from Lyn-deficient, Fyn-deficient, Lyn/Fyn double deficient or wild-type control mice [4]. After sacrifice of the mice by CO_2_ inhalation, bone marrow cells were cultured in RPMI-1640 containing 16% fetal calf serum, glutamax, non-essential amino acids, streptomycin and penicillin, and supplemented with 20 ng/ml interleukin-3 and 20 ng/ml Stem Cell Factor (both from Peprotech, Rocky Hill, NJ). After four weeks in culture more than 95% of the cells were mast cells as assessed by FcεRI and c-kit expression and toluidine blue staining.

### Reconstitution experiments

A lentiviral approach was used that was essentially as previously described [40]. The lentiviral vectors carried Lyn kinase or the catalytically inactive form of Lyn (K172R). The forward and reverse primer sequences used to insert a point mutation in the kinase domain of Lyn (Lyn K172R) were as follows: 5′-GTGGCTGTGAGGACCCTCA-3′ (forward), 5′-CTTGAGGGTCCTCACAGCC-3′ (reverse).

### Cell stimulation and lysis

One million RBL-2H3 cells, variants or transfectants in 2 ml or BMMC at 1×10^6^/ml were plated overnight with or without anti-DNP IgE ascitic fluid (1∶10^3^ dilution for RBL-2H3 cells and 1∶10^2^ dilution for BMMC). After several washes in Tyrode’s solution (Hepes 10 mM pH 7.3, NaCl 135 mM, KCl 5 mM, glucose 5.6 mM, CaCl_2_ 1.8 mM, MgCl_2_ 1 mM, BSA 0.5 mg/ml), cells (1×10^6^/ml RBL-2H3 cells, variants or transfectants, or 2×10^6^/ml BMMC) were stimulated at 37°C for the time indicated with 1 µg/ml DNP-HSA for RBL-2H3 cells, variants or transfectants and with 0.1 µg/ml DNP-HSA for BMMC.

For stimulation of FcαRI transfectants (wild-type and R209L mutated proteins), cells were first incubated with 10 µg/ml F(ab’)2 fragment of the anti-FcαRI monoclonal antibody A77 [41] for 1 hr at 4°C in Tyrode’s buffer. Cells were then washed twice and stimulated for the indicated time at 37°C with 20 µg/ml F(ab’)2 fragment of rabbit anti-mouse IgG antibody.

In all experiments, the supernatant was recovered for control of degranulation and cells were washed in ice-cold PBS. Cells were lysed on ice in 200 µl lysis solution (Hepes 50 mM pH 7.2, 50 mM NaF, 50 mM NaCl, 1 mM Na_3_VO_4_, 1% Triton X-100, 0.1% SDS, 10 µg/ml leupeptin, 10 µg/ml aprotinin). After 10 min on ice, the wells were scraped, their content transferred to microtubes and the soluble cell lysates were recovered following centrifugation at 14,000 g for 10 minutes at 4°C for immunoblotting or immunoprecipitation. For co-immunoprecipitation purposes, 1×10^7^ cells were lysed in 500 µl lysis buffer containing 0.5% Triton X-100.

### Imumunoprecipitation and immunoblotting

Proteins in the soluble cell lysates were immunoprecipitated with either Sepharose-4B (Pharmacia, Uppsala, Sweden) coupled antibodies (for 129.2, and control IgG) or with anti-mouse PLSCR1 monoclonal antibody 1A8 adsorbed to protein G beads (Pharmacia, Uppsala, Sweden), by incubation in a rotating wheel for 2 hours (hr) at 4°C. Beads were washed 6 times with 1 ml ice-cold lysis buffer and material was eluted by boiling for 5 min in Laemmli sample buffer [42].

Immunoblots were performed after resolution of proteins by SDS-PAGE and their transfer onto PVDF membranes. After blocking non specific sites by incubation for 1 hr in 10 mM Tris pH 7.2 containing 150 mM NaCl, 0.05% Tween 20 and 4% BSA, membranes were incubated with the desired first antibody for 1 hr, washed 3 times for 10 min, incubated with the relevant secondary antibody and washed 3 times for 10 min. Blots were revealed by incubation in SuperSignal West Pico solution from Pierce (Rockford, IL) and exposure to X-OMAT films from Kodak (Rochester, NY). In some cases, membranes were stripped by several washes in methanol and reblotted with control antibodies. Blots were quantified by densitometry using the National Institutes of Health Software *Image J 1.46r* after scanning of the films. In the case of PLSCR1 the band corresponding to phosphorylated PLSCR1 was normalized to the total PLSCR1 protein recovered in the precipitates for each experimental point. Fold phosphorylation is the ratio of the corrected value of phospho-PLSCR1 obtained with stimulated cells to that obtained with the non-stimulated cells.

### Degranulation measurements

Degranulation was assessed by measurement of the release of the granule marker β-hexosaminidase as described [43].

### Calcium measurement

RBL-2H3 cells were trypsinized and washed two times in culture medium. Cells were then sensitized in the presence 1∶10^3^ dilution of anti-DNP IgE ascitic fluids for two hours in a 37°C waterbath with frequent agitation and washed in Tyrode’s buffer with or without Ca^++^ and Mg^++^. Cells were incubated or not with 40 µM of the intracellular calcium chelator BAPTA-AM (Calbiochem, La Jolla, CA) for 15 minutes at 37°C protected from light. Treated cells were washed two times in Tyrode’s buffer with or without Ca^++^ and Mg^++^. All cells were resuspended at 1×10^6^/ml in Tyrode’s buffer with or without Ca^++^ and Mg^++^. Cells were loaded with 5 µM Fura-2-AM (Calbiochem, La Jolla, CA) for 30 minutes at 37°C protected from light, washed two times in Tyrode’s buffer with or without Ca^++^ and Mg^++^, and resuspended at 1.5×10^6^/ml. The fluorescence of free intracellular Ca^++^, after stimulation of the cells with 1 µg/ml DNP-HSA, was measured at 37°C using two excitation wavelengths at 340 and 380 nm and an emission wavelength of 510 nm in an Hitachi H-2000 spectrofluorimeter (Sciencetec, Les Ulis, France). Ca^++^ concentrations were calculated using a Kd of 224 nM for the interaction between Fura-2 and Ca^++^.

### Statistical analyses

All experiments were conducted at least three times (see figure legends). Statistical analyses were performed using GraphPad Prism 5.0 as indicated in figure legends.

## Results

### IgE-mediated PLSCR1 tyrosine phosphorylation is dependent on the FcRγ chain

**Figure 1:**
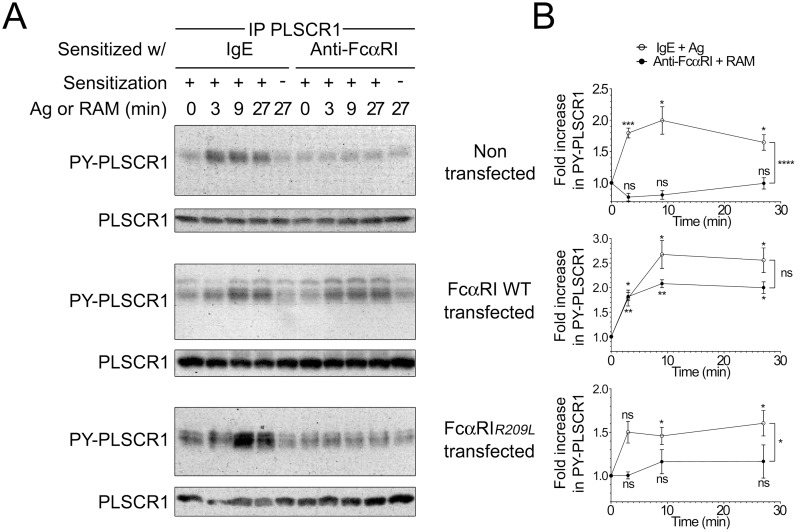
FcεRI-mediated PLSCR1 tyrosine phosphorylation depends on the FcRγ chain. (A) Non-transfected, FcαRI-transfected or FcαRI*_R209L_*-transfected RBL-2H3 cells were sensitized for 1 hr with anti-DNP IgE or F(ab’)2 fragment of the anti-FcαRI monoclonal antibody A77, as indicated. After washes, IgE-sensitized cells were stimulated with specific antigen (*Ag*), whereas A77-F(ab’)2-sensitized cells were stimulated with F(ab’)2 fragment of rabbit anti-mouse IgG (*RAM*) for the indicated time. PLSCR1 in cell lysates was immunoprecipitated with anti-rat PLSCR1 monoclonal antibody 129.2 (*IP PLSCR1*). Eluates were analyzed by immunoblotting with anti-phosphotyrosine monoclonal antibody to detect PY-PLSCR1 and, after stripping of the membranes, with 129.2 to detect total PLSCR1, as indicated. (B) Quantification of PLSCR1 tyrosine phosphorylation relative to immunoprecipitated PLSCR1. Shown are the fold increases relative to basal PLSCR1 phosphorylation. Two-way ANOVA was used to compare the two kinetics observed for each cell line (bracket); and two-tailed unpaired student t test was used to compare stimulated conditions to unstimulated condition. Data are presented as mean ± s.e.m. of at least three independent experiments. ns: not significant; *: p<0.05; **: p<0.01; ***: p<0.001.

The tetrameric form of FcεRI is composed of one FcεRIα chain responsible for IgE binding, and of the signal transducing chains FcεRIβ and a dimer of FcRγ (αβ(γ)_2_). The FcRγ chain is also mandatory for FcεRI cell membrane expression. We first examined whether this chain was required for the signal leading to PLSCR1 tyrosine phosphorylation. The FcRγ chain is shared by many Fc receptors [44] including the receptor for IgA (FcαRI) [45], [46]. Interestingly, this receptor cannot associate with the FcRγ chain when an arginine-to-leucine (R209L) mutation is introduced in its transmembrane domain but is readily expressed at the plasma membrane [39]. Since mast cells do not express FcαRI, we took advantage of RBL-2H3 rat mast cells that had been transfected with wild-type or mutant (R209L) FcαRI. Of note, these transfectants expressed comparable levels of FcαRI at the plasma membrane and engagement of wild-type FcαRI induced degranulation of the transfectants whereas aggregation of of the R209L mutant did not ([47] *and data not shown*). As in the parental cell line, aggregation of FcεRI in these transfectants resulted in PLSCR1 tyrosine phosphorylation (figure 1). Yet, whereas tyrosine phosphorylation of PLSCR1 was readily observed after aggregation of wild-type FcαRI, no such phosphorylation could be detected after engagement of the FcαRI*_R209L_* mutated form of FcαRI (figure 1). We conclude that PLSCR1 phosphorylation required the FcRγ chain.

### IgE-mediated PLSCR1 tyrosine phosphorylation depends on Lyn

**Figure 2:**
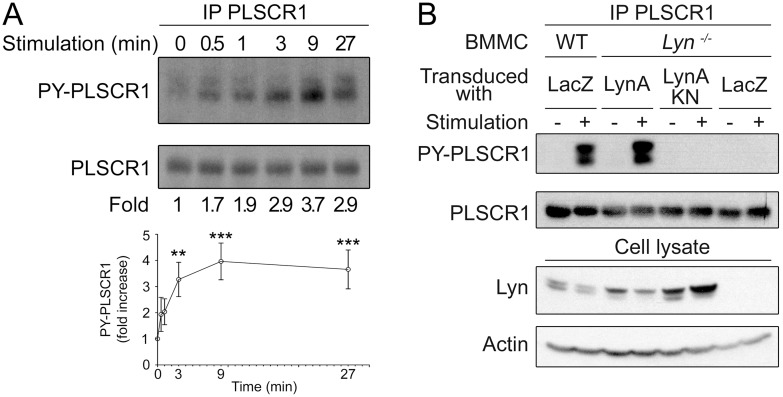
Time- and Lyn-dependency of FcεRI-mediated PLSCR1 tyrosine phosphorylation. (A) Time-dependent FcεRI-mediated PLSCR1 tyrosine phosphorylation in WT mouse BMMC. IgE-sensitized cells were stimulated for the indicated length of time with antigen. PLSCR1 was immunoprecipitated from cell lysates and tyrosine phosphorylated PLSCR1 (PY-PLSCR1) was analyzed by immunoblotting with anti-phosphotyrosine antibody (4G10). After stripping of the membrane, total PLSCR1 was analyzed by immunoblotting with anti-mouse PLSCR1 1A8 antibody. Lower panel: quantification. Statistical analysis was done by a one-way ANOVA followed by a Tukey’s multiple comparison test. Data are presented as mean ± s.e.m. of six independent experiments. **: p<0.01; ***: p<0.001. (B) FcεRI-mediated PLSCR1 tyrosine phosphorylation is dependent on Lyn. Wild-type or Lyn−/− BMMC were transduced either with empty vector (*LacZ*), LynA containing vector (*LynA*) or dead-kinase LynA (*LynAKN*). After reconstitution, IgE-sensitized cells were stimulated for 30 minutes with antigen. Upper panels: PLSCR1 was immunoprecipitated from cell lysates and tyrosine phosphorylated PLSCR1 (PY-PLSCR1) was analyzed by immunoblotting with anti-phosphotyrosine antibody. After stripping of the membrane, total PLSCR1 was analyzed by immunoblotting with anti-mouse PLSCR1 1A8 antibody. Lower panels: Controls for the presence of Lyn in the reconstituted cells were performed by immunoblotting cell lysates with anti-Lyn antibody and with anti-actin antibody for loading control.

It is well established that Lyn [2] and Fyn [4], both Src-family kinase members, function immediately downstream of the receptor. To determine which one of these kinases was important for PLSCR1 tyrosine phosphorylation, we used bone marrow-derived mast cells (BMMC) from Lyn-deficient and from Fyn-deficient mice. As a first step, FcεRI-dependent tyrosine phosphorylation of PLSCR1 was examined in wild-type BMMC. An increased phosphorylation was observed as soon as 30 sec after FcεRI engagement and reached a plateau after 9 min stimulation (Figure 2A) confirming that the phosphorylation observed in RBL-2H3 rat mast cells was not the result of the tumoral phenotype of this cell line and that it was not restricted to rat mast cells. Therefore in all BMMC experiments, subsequent analyses were performed after at least 9 minutes of stimulation. As in RBL-2H3 cells [7] phospho-PLSCR1 migrated as a heterogeneous band between 37 and 48 kDa (Figure 2A). We first assessed the involvement of Lyn in the PLSCR1 tyrosine phosphorylation by using BMMC from *Lyn* ^−/−^ mice. Interestingly, no increase in tyrosine phosphorylation of PLSCR1 was observed in these cells after FcεRI aggregation (Figure 2B). Reconstitution of the *Lyn* ^−/−^ cells with kinase-sufficient Lyn restored PLSCR1 tyrosine phosphorylation. By contrast, reconstitution with kinase-deficient Lyn was unable to restore this phosphorylation. These results show that Lyn is mandatory and its kinase activity is required for FcεRI-dependent increase in PLSCR1 phosphorylation.

### IgE-mediated PLSCR1 tyrosine phosphorylation is negatively regulated by Fyn

**Figure 3:**
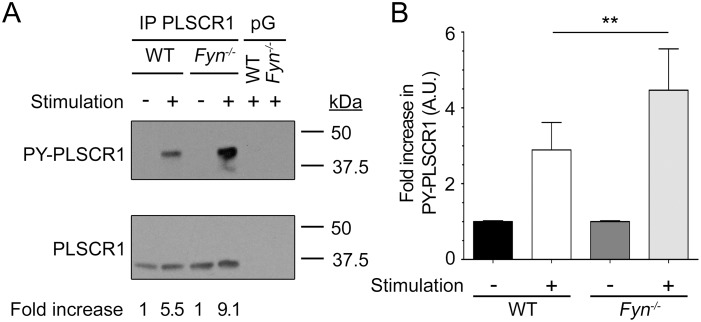
FcεRI-mediated PLSCR1 tyrosine phosphorylation is negatively regulated by Fyn. (A) Five million IgE-sensitized BMMC from wild-type (WT) or Fyn knock-out (*Fyn* ^−/−^) mice were stimulated or not with antigen for 10 min. BMMC cell lysates were subjected to immunoprecipitation with anti-mouse PLSCR1 mAb 1A8 or protein G beads alone (pG), and eluates were analyzed by immunoblotting with anti-phosphotyrosine (upper panel) and, after stripping, anti-muPLSCR1 (lower panel) monoclonal antibodies. Fold increase in phosphorylation corresponds to the ratio of the value of phospho-PLSCR1 obtained for stimulated and non-stimulated cells for each condition normalized with the corresponding value of recovered total PLSCR1. (B) Quantification. Statistical analysis was done by a one-way ANOVA followed by a two tailed paired student t test. Data are presented as mean + s.e.m. of four independent experiments. **: p<0.01.

**Figure 4:**
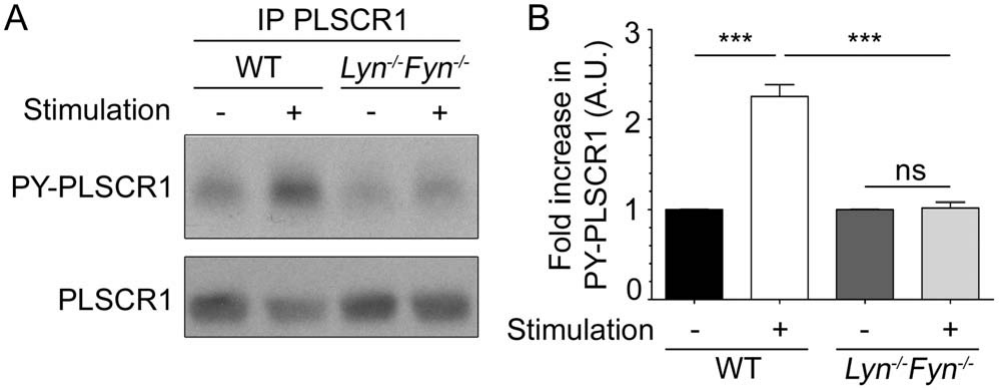
The Fyn-dependent negative regulation of FcεRI-mediated PLSCR1 tyrosine phosphorylation targets Lyn-dependent PLSCR1 phosphorylation. (A) Five million IgE-sensitized BMMC from wild-type (WT) or Lyn/Fyn-double deficient (*Lyn* ^−/−^ *Fyn* ^−/−^) mice were stimulated or not with antigen for 10 min and PLSCR1 was immunoprecipitated from the lysates with anti-muPLSCR1 antibody 1A8. Tyrosine phosphorylated PLSCR1 (PY-PLSCR1) recovered from the lysates was detected by immunoblotting with anti-phosphotyrosine antibody and, after stripping of the membrane, the total amount of recovered muPLSCR1 was analyzed. (B) Quantification. Statistical analysis was done by a one-way ANOVA followed by a Tukey’s multiple comparison test. Data are presented as mean + s.e.m. of three independent experiments. ns: not significant; ***: p<0.001.

**Figure 5:**
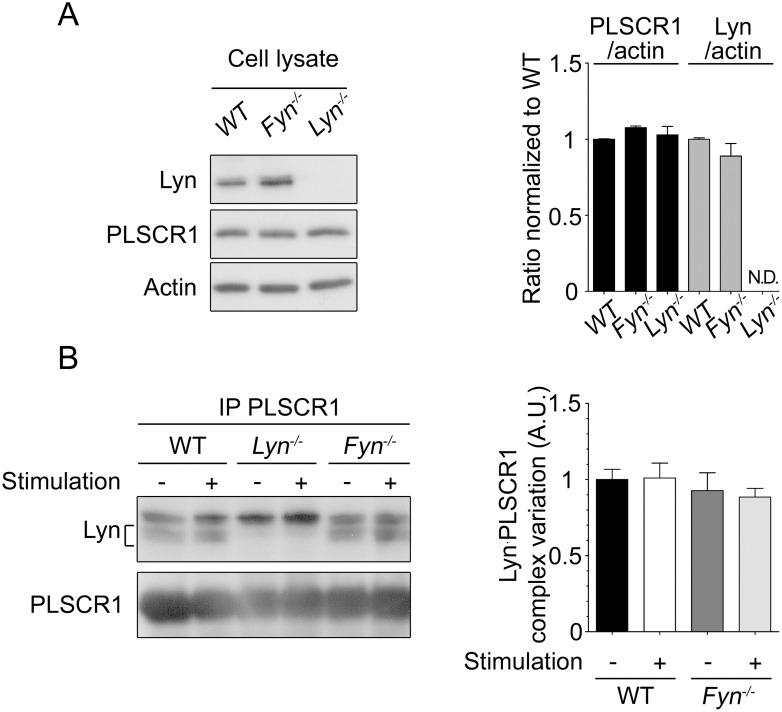
Lyn: PLSCR1 association is not modulated by Fyn in mast cells. A) Quantification of PLSCR1 and Lyn relative to actine in wild-type (WT), Lyn^−/−^ and Fyn^−/−^ BMMC. Left: representative immunoblots for the indicated proteins from one analysis. Right: relative quantifications of three independent experiments. The relative amount of the indicated protein was standardized as 1 in WT BMMC, and served as reference for all other relative quantifications. N.D., not detected. Data are presented as mean + s.e.m. One-way ANOVA analysis showed no significant difference. B) Sensitized BMMC from wild-type (WT), from Fyn-deficient (*Fyn* ^−/−^) and from Lyn-deficient (*Lyn* ^−/−^) mice were stimulated or not stimulated with antigen for 10 min. BMMC cell lysates were immunoprecipitated with anti-mouse PLSCR1 mAb 1A8-coupled beads (IP PLSCR1). Eluates were analyzed by immunoblotting with anti-Lyn antibodies (Lyn) and, after stripping, with anti-muPLSCR1 monoclonal antibody (PLSCR1). Left: one experiment representative of four. Bracket: Lyn doublet. The upper band is the heavy chain of the immunoprecipitating antibody. Right: Quantification of the amounts of Lyn co-immunoprecipitated with PLSCR1. Data were normalized to values obtained with WT unstimulated BMMC. Data are presented as mean + s.e.m. of four independent experiments. One-way ANOVA analysis showed no significant difference.

Surprisingly, the increased tyrosine phosphorylation of PLSCR1 after FcεRI engagement was higher in Fyn-deficient BMMC (Figure 3), revealing that Fyn is a negative regulator for this event. This more robust increase in PLSCR1 phosphorylation was also dependent on Lyn. No increased PLSCR1 phosphorylation was observed in BMMC deficient for both Lyn and Fyn (Figure 4) suggesting that Fyn impedes the Lyn-dependent PLSCR1 phosphorylation rather than targeting an altogether different pathway. This led us to examine whether Fyn deficiency had an impact on the expression of Lyn or of PLSCR1, or on their association. There was no difference in Lyn or PLSCR1 expression between wild-type and Fyn-deficient BMMC (Figure 5A). As well, co-immunoprecipitation experiments demonstrated that the absence of Fyn did not result in a detectable increase in Lyn association with PLSCR1 (Figure 5B), although it confirmed in BMMC an association between Lyn and PLSCR1 that was not modulated by FcεRI aggregation as we previously reported in RBL-2H3 cells [33]. We conclude that Lyn-dependent and Fyn-dependent signals converge as antagonists in the regulation of FcεRI-mediated PLSCR1 tyrosine phosphorylation.

### IgE-mediated PLSCR1 tyrosine phosphorylation depends on Syk

**Figure 6:**
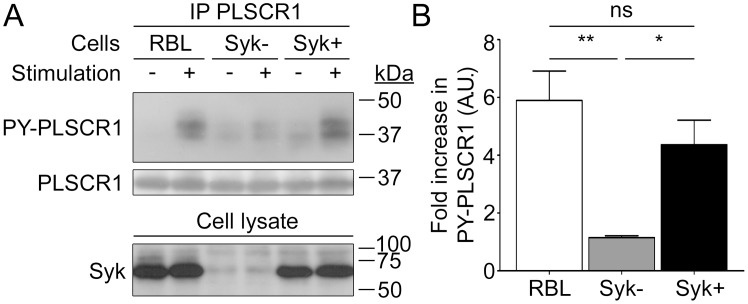
FcεRI-mediated PLSCR1 tyrosine phosphorylation is dependent on Syk. (A) One million IgE-sensitized RBL-2H3 cells (RBL) and Syk-deficient RBL-2H3 cells before (Syk–) or after (Syk+) reconstitution with Syk, were stimulated for 30 minutes with antigen. PLSCR1 was immunoprecipitated from cell lysates with anti-rat PLSCR1 monoclonal antibody 129.2. Tyrosine phosphorylated PLSCR1 (PY-PLSCR1) in eluates was detected by immunoblotting with phosphotyrosine-specific antibodies and, after stripping of the membrane, PLSCR1 was detected by immunoblotting with 129.2. Lower panel: the presence and absence of Syk was confirmed in cell lysates by immunoblotting. (B) Quantification of the increase in PLSCR1 tyrosine phosphorylation after cell stimulation relative to PLSCR1 basal phosphorylation. Statistical analysis was done by a one-way ANOVA followed by a Tukey’s multiple comparison test. Data are presented as mean + s.e.m. of four independent experiments. ns: not significant; *: p<0.05; **: p<0.01.

Syk is a tyrosine kinase that is activated downstream of Lyn [1] and that coprecipitates with PLSCR1 [38]. Therefore, we used a variant of RBL-2H3 that is deficient in Syk [38] to evaluate its involvement in PLSCR1 phosphorylation. Aggregation of FcεRI on these cells resulted in no significant increase in tyrosine phosphorylation of PLSCR1 (Figure 6). Reconstitution of these cells with Syk restored in great part the FcεRI-induced tyrosine phosphorylation of PLSCR1 (Figure 6). These data demonstrate that Syk is required for optimal FcεRI-dependent phosphorylation of PLSCR1.

### IgE-mediated PLSCR1 tyrosine phosphorylation is partially dependent on intracellular calcium mobilization

**Figure 7:**
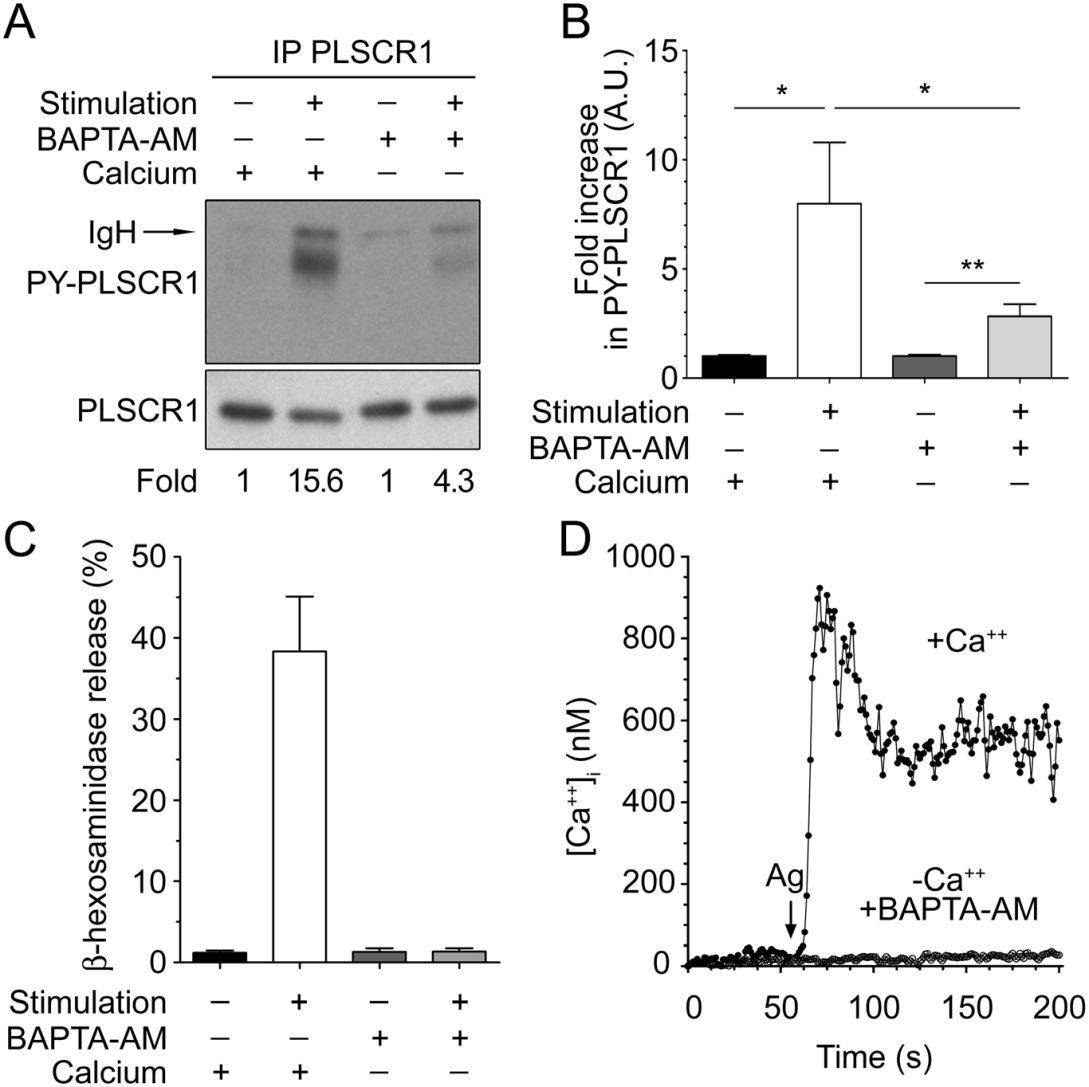
FcεRI-mediated PLSCR1 tyrosine phosphorylation is partially dependent on calcium. (A) Phosphorylation of PLSCR1 in the absence of intra- and extra-cellular calcium. RBL-2H3 cells were stimulated or not stimulated with IgE and antigen for 30 min in the presence or absence of calcium and of the intracellular calcium chelator BAPTA-AM as indicated and as described in the Materials and Methods Section. RBL-2H3 cell lysates were immunoprecipitated with anti-rat PLSCR1 mAb 129.2, and tyrosine phosphorylated PLSCR1 (PY-PLSCR1) in the eluates was analyzed in immunoblotting with anti-phosphotyrosine antibodies. After stripping of the membrane, the presence of total PLSCR1 was analyzed by immunoblotting with anti-rat PLSCR1 monoclonal antibodiy 129.2. Quantification of the PLSCR1 phosphorylation was performed using the NIH Image J software. Fold phosphorylation corresponds to the ratio of the value of phospho-PLSCR1 obtained for stimulated and non-stimulated cells (*Fold*, see Materials and Methods section) for each condition. One experiment representative of three is shown. IgH: Heavy chain of immunoprecipitating antibody. (B) Quantification. Statistical analysis was done by a one-way ANOVA followed by a two tailed paired student t test. Data are presented as mean + s.e.m. of three independent experiments. *: p<0.05; **: p<0.01. (C) Degranulation. The supernatants of the cells shown in the panel (A) were tested for the percent of β-hexosaminidase released. (D) Fluorescence measurement of intracellular calcium of sensitized RBL-2H3 cells loaded with Fura-2-AM and stimulated as in (A) with antigen (Ag) in the presence (+Ca^++^) and absence (−Ca^++^ + BAPTA-AM) of intra- and extra-cellular calcium. This experiment is representative of three.

Calcium mobilization occurs downstream of Lyn and of Syk activation in FcεRI-activated mast cells [4], [38], and FcεRI engagement induces both early (calcium independent) and late (calcium dependent) tyrosine phosphorylations [48]. PLSCR1 has an EF-hand-like domain allowing calcium binding that results in a conformational change of the molecule [49]–[51]. In addition, calcium ionophores can induce PLSCR1 tyrosine phosphorylation [52]. Therefore, we examined whether FcεRI-dependent phosphorylation of PLSCR1 is modulated by calcium mobilization that occurs in response to activation of this receptor. To that effect, RBL-2H3 cells were activated in the presence extracellular calcium, or in cells loaded with the intracellular calcium-chelator BAPTA-AM and suspended in calcium-free medium. In the absence of calcium (and in the presence of BAPTA-AM), aggregation of FcεRI induced a detectable increase in tyrosine phosphorylation of PLSCR1. Yet, this increase amounted to approximately 25% of that observed after FcεRI aggregation in the presence of calcium (figure 7A). Under these conditions, and as previously reported [53], no degranulation (figure 7B) and no detectable calcium signal (figure 7C) were observed. Although one cannot completely exclude that infinitesimal or highly localised variations of free cytosolic calcium could account for the residual tyrosine phosphorylation of PLSCR1 in the absence of external calcium and in the presence of BAPTA, our data strongly indicate collectively that IgE-mediated PLSCR1 phosphorylation involves calcium-dependent and calcium-independent signals.

## Discussion

**Figure 8:**
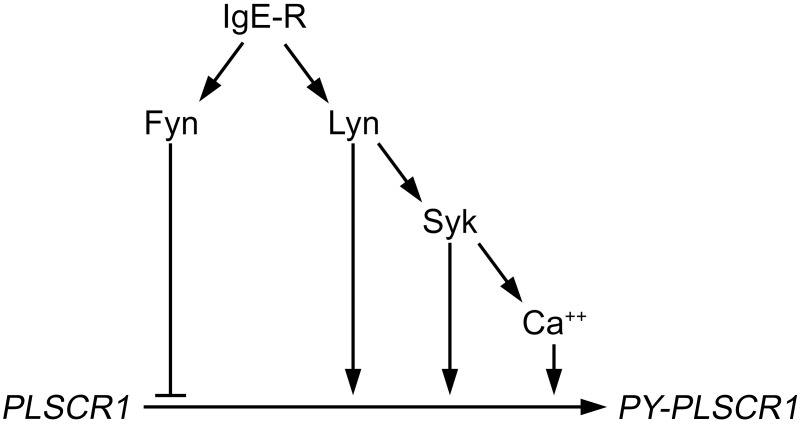
Complex regulation of FcεRI-dependent tyrosine phosphorylation of PLSCR1. Based on the data presented herein, we propose that tyrosine phosphorylation of PLSCR1 following FcεRI engagement is regulated on at least three levels in mast cells. Positive regulation is mediated by the Lyn-dependent pathway, whereas negative regulation is mediated by the Fyn-dependent pathway. After FcεRI aggregation, PLSCR1 can be phosphorylated on tyrosine directly either by Lyn or by Syk and indirectly as a result of Lyn/Syk-dependent activation of subsequent calcium mobilization. The other activation pathway initiated by FcεRI, that is dependent on Fyn, negatively regulates tyrosine phosphorylation of PLSCR1. Whether it acts by directly modulating Lyn-mediated or calcium-dependent tyrosine phosphorylation of PLSCR1, by controlling the PLSCR1 cellular localization required for its optimal phosphorylation by the Lyn pathway or by promoting its dephosphorylation is still unresolved.

We previously reported that PLSCR1 is phosphorylated on tyrosine after aggregation of FcεRI expressed on the RBL-2H3 rat mast cell line [7]. Background levels of PLSCR1 tyrosine phosphorylation before receptor engagement varied from experiment to experiment but could be seen in all cases upon sufficient exposure time of immunoblots. This was true not only for RBL cells, but also for BMMC. We believe that these variations might be related to variations in batch-to-batch cell cultures as we routinely observed variations in cell growth intensity and in degranulation responses of BMMC. However, a statistical increase in PLSCR1 tyrosine phosphorylation was reproducibly observed after aggregation of the receptor in WT BMMC. The data presented here demonstrate that this phosphorylation is subject to regulation that involves calcium mobilization as well as both positive and negative regulatory mechanisms mediated by Lyn and Syk, and Fyn, respectively (summarized in Figure 8). These data suggest that the phosphorylation of PLSCR1 is pivotal to the cross-interaction of the Lyn- and Fyn-initiated signaling pathways. Whereas initial analyses of FcεRI-dependent signaling pathways concentrated on defining specific effectors for each pathway, it becomes increasingly evident that multiple signals converge and that cross-talk of signals is key for an integrated cellular response. Example of this is provided by the tyrosine kinase Csk and by the adaptor Cbp that are recruited by Lyn and that negatively regulate the Fyn signaling in FcεRI-mediated mast cell activation [6]. Therefore, whereas Lyn can negatively regulate Fyn-initiated signals [6], herein we report that, conversely, Fyn can negatively regulate at least some Lyn-initiated signals, demonstrating that both pathways have the capacity to control each other. Thus, particular substrates could function at such crossroads promoting integration of signaling pathways and allowing fine-tuning and regulation. Interestingly, we previously demonstrated that PLSCR1 acts as an amplifier of the LAT-PLCγ-calcium axis thus modulating degranulation and VEGF production [33]. This axis depends on the Lyn-initiated signaling pathway. The association between Lyn and PLSCR1 is reminiscent of the association between PLSCR1 and Src in EGF receptor signaling that potentiates Src kinase activity [20], [21]. Our present study demonstrates that whereas PLSCR1 can modulate the Lyn initiated pathway, this pathway controls PLSCR1 tyrosine phosphorylation, revealing a particular partnership between PLSCR1 and Lyn in FcεRI-induced mast cell activation.

The absolute requirement of Lyn and Syk is demonstrated by the absence of FcεRI-dependent increase in PLSCR1 phosphorylation in mast cells deficient for either kinase. Of note, absence of either kinase abolishes the mobilization of calcium [4], [38]. Yet, we observed a residual, but significant, increase in tyrosine phosphorylation of PLSCR1 in the absence of calcium after FcεRI aggregation. This indicates that PLSCR1 could be phosphorylated on multiple tyrosines (calcium-dependent and calcium-independent), which would be in agreement with the heterogeneous molecular weight observed for phospho-PLSCR1 in mast cells, although the extent of this heterogeneity varies somehow from experiment to experiment for reasons that remain so far unclear. The increase in PLSCR1 tyrosine phosphorylation observed in the presence of calcium may be due to its phosphorylation on additional phosphorylation sites accessible after the conformational change induced by calcium [49], [50]. Alternatively, new molecules of PLSCR1 could be recruited for tyrosine phosphorylation by the same or other kinases.

That Lyn and Syk could be directly involved in the phosphorylation of PLSCR1 is supported by several observations. Lyn and Syk were found to physically interact with PLSCR1 [33]. In addition, the FcεRI-dependent tyrosine phosphorylation of PLSCR1 was fully ablated in the absence of FcRγ, and partially in the absence of calcium, mapping the occurrence of the residual increase in the phosphorylation of PLSCR1 upstream of the calcium signal but downstream of the FcRγ chain, i.e. where Lyn and Syk are thought to function in IgE-mediated signaling [1]. Moreover, tyrosine phosphorylation of PLSCR1 was recovered when *Lyn^−/−^* BMMC and Syk-deficient RBL-2H3 cells were reconstituted with kinase competent Lyn or Syk, respectively, demonstrating that the defect in PLSCR1 phosphorylation was not due to aberrant maturation of these cells but to a kinase-related defect in FcεRI signaling. After FcεRI aggregation no significant increase in Lyn association with PLSCR1 was observed indicating that tyrosine phosphorylation of PLSCR1 by Lyn is not solely regulated by the interaction between both proteins but might be also regulated by Lyn activation. Previous studies have demonstrated that PLSCR1 also interacts with the prototypic kinase p60c-src within the EGF receptor signaling pathways [20], [21], thus serving as a substrate for p60c-src and, in turn, amplifying the activation of this kinase [20]. These data together with the ones collected herein, suggest a preferred connection between PLSCR1 and this family of tyrosine kinases that might be due to their common localization at the plasma membrane and particularly in lipid rafts [21], [33].

This preferred connection is also highlighted by the negative regulation of PLSCR1 tyrosine phosphorylation by Fyn, another member of the Src family. This was revealed by the more robust increase in FcεRI-dependent phosphorylation of PLSCR1 in Fyn-deficient BMMC. The increased phosphorylation of PLSCR1 was not due to an increased calcium signal because Fyn deficiency does not increase this signal [4]. Neither was it due to an overall increase in tyrosine phosphorylation of cellular proteins since tyrosine phosphorylation of proteins in whole cell lysates from activated cells is significantly lower in Fyn-deficient BMMC compared to their wild-type counterparts ([4] and *data not shown*).

The data presented here extend our original observation that rat PLSCR1 is phosphorylated on tyrosine after FcεRI aggregation in the RBL-2H3 mast cell line [7] to non-tumoral mast cells derived in culture (BMMC) and to another species. Furthermore, the demonstration that it is initiated by the FcRγ chain and by another FcRγ-associated receptor (the transfected FcαRI) suggests that other Fc receptors should be able to promote the tyrosine phosphorylation of PLSCR1. The complexity of the mechanisms regulating PLSCR1 tyrosine phosphorylation suggests that this phosphorylation might play an important role in the regulation of PLSCR1 amplifier function. Studies by others have reported that there is no correlation between mast cell degranulation, phosphatidylserine externalization and tyrosine phosphorylation of PLSCR1 when comparing mast cells stimulated through FcεRI, Thy-1 and calcium ionophores [52]. The evidence presented here suggesting that PLSCR1 is phosphorylated on multiple tyrosines raises the possibility that positive regulation of degranulation by PLSCR1 may be associated with its phosphorylation on particular tyrosines whereas phosphorylation of other tyrosines may be involved in down-regulation of PLSCR1 function as is known for many receptors. Studies are underway to clarify this question.
